# Thermocatalytic Hydrogen Production Through Decomposition of Methane-A Review

**DOI:** 10.3389/fchem.2021.736801

**Published:** 2021-10-25

**Authors:** Gowhar A. Naikoo, Fareeha Arshad, Israr U. Hassan, Musallam A. Tabook, Mona Z. Pedram, Mujahid Mustaqeem, Hassina Tabassum, Waqar Ahmed, Mashallah Rezakazemi

**Affiliations:** ^1^ Department of Mathematics and Sciences, College of Arts and Applied Sciences, Dhofar University, Salalah, Oman; ^2^ Department of Biochemistry, Aligarh Muslim University, Aligarh, India; ^3^ College of Engineering, Dhofar University, Salalah, Oman; ^4^ Mechanical Engineering-Energy Division, K. N. Toosi University of Technology, Tehran, Iran; ^5^ Institute of Physics, Academia Sinica, Taipei, Taiwan; ^6^ Department of Chemistry, National Taiwan University, Taipei, Taiwan; ^7^ Department of Chemical and Biological Engineering, State University of New York at Buffalo, Buffalo, NY, United States; ^8^ School of Mathematics and Physics, College of Science, University of Lincoln, Lincoln, United Kingdom; ^9^ Faculty of Chemical and Materials Engineering, Shahrood University of Technology, Shahrood, Iran

**Keywords:** hydrogen production, thermocatalytic methane decomposition, energy, catalysts, catalytic regeneration

## Abstract

Consumption of fossil fuels, especially in transport and energy-dependent sectors, has led to large greenhouse gas production. Hydrogen is an exciting energy source that can serve our energy purposes and decrease toxic waste production. Decomposition of methane yields hydrogen devoid of CO_x_ components, thereby aiding as an eco-friendly approach towards large-scale hydrogen production. This review article is focused on hydrogen production through thermocatalytic methane decomposition (TMD) for hydrogen production. The thermodynamics of this approach has been highlighted. Various methods of hydrogen production from fossil fuels and renewable resources were discussed. Methods including steam methane reforming, partial oxidation of methane, auto thermal reforming, direct biomass gasification, thermal water splitting, methane pyrolysis, aqueous reforming, and coal gasification have been reported in this article. A detailed overview of the different types of catalysts available, the reasons behind their deactivation, and their possible regeneration methods were discussed. Finally, we presented the challenges and future perspectives for hydrogen production via TMD. This review concluded that among all catalysts, nickel, ruthenium and platinum-based catalysts show the highest activity and catalytic efficiency and gave carbon-free hydrogen products during the TMD process. However, their rapid deactivation at high temperatures still needs the attention of the scientific community.

## Introduction

**FIGURE 1:**
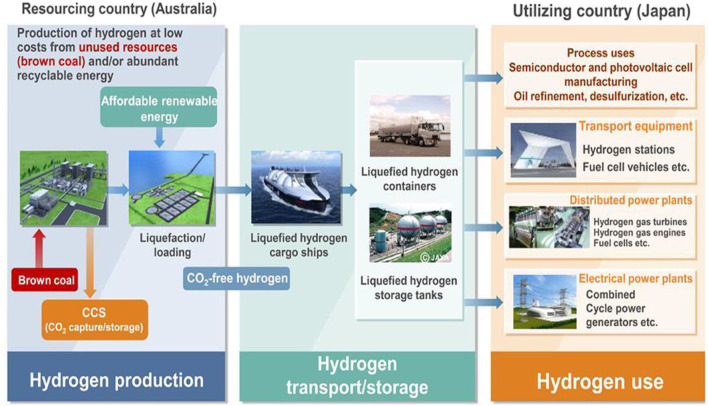
Concept of CO_2_ free hydrogen chains for Japan. Reproduced with permission from references Bockris (2013), Eljack and Kazi (2021), copyright@2021 (Frontiers).

Hydrogen (H_2_) is one of the fundamental energy storage elements that are found in most chemical compounds including water and hydrocarbons. The future of world energy is dependent on hydrogen and therefore, the term “hydrogen economy” was coined that points out that highly pure hydrogen will have to be produced to sustain the hydrogen economy (Bockris, 2013; Eljack and Kazi, 2021). The worldwide production of hydrogen is ∼70 million tons per year and is majorly produced from coal and gas. According to Energy Information Administration, about 7% of natural gas is responsible for hydrogen production (Hydrogen Council, 2017). However, a very minute amount (∼6%) of the hydrogen produced satiates the demand of pure hydrogen (Bockris, 2013; Eljack and Kazi, 2021). Hydrogen being a cleaner source of energy will replace the traditional fuel sources and help conserve the environment better. For hydrogen production, fossil fuels are the primary source owing to their inexpensive availability, easy access, and simple apparatus requirements (El-Shafie et al., 2019). However, their ability to cause environmental pollution is a major concern. Various methods have been explored for the production of hydrogen however, the steam reforming and coal gasification (Srilatha et al., 2017) methods have exhibited the dominance because of their exciting results. Depending upon the feedstock used, different pathways are adopted to produce concentrated hydrogen (Ghavam et al., 2021). Hydrogen is also derived from renewable sources like the naturally available wind and sun (Carroquino et al., 2019). Hydrogen is also produced using elements like uranium among others (Chen et al., 2019a). Thus the different methods used in hydrogen production give varying amounts of hydrogen and in turn varying amounts of byproducts. For instance, hydrogen produced via fossil fuels accounts for 830 million tons of annual carbon dioxide that is about ∼2% of carbon dioxide emission worldwide (Baharuddin et al., 2021; Singla et al., 2021). As per the International Energy Agency, over 0.36 million tons of hydrogen produced was of low carbon in 2019 (Karaca and Dincer, 2020). This means that clean hydrogen production is about 0.52% of hydrogen produced worldwide and at this rate, 7.92 million tons of low carbon hydrogen will be produced yearly by 2030 (Karaca and Dincer, 2020). In order to decarbonize transport sector, Japan has formulated a hydrogen plan in 2014. This is to reduce the dependence on fossil fuels and decrease the resultant greenhouse emissions (Bockris, 2013; Eljack and Kazi, 2021) as shown in Figure 1.

**FIGURE 2:**
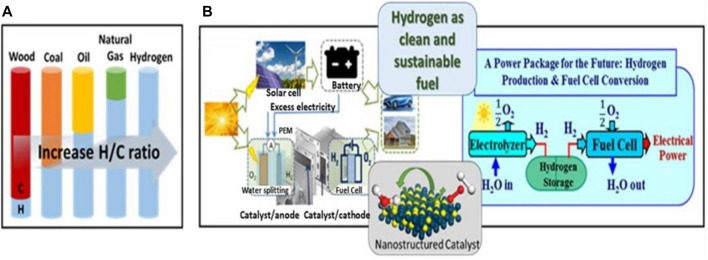
**(A)** An illustration of the evolution of fuels in terms of hydrogen to carbon ratio. **(B)** Illustrations of a dual cell functioning as an electrolysis water-splitting cell for hydrogen production from solar energy and a fuel cell for the conversion of hydrogen to electricity, highlighting the sustainable power package of the future and the role of catalysis. Adapted from reference Wang et al. (2021a) Copyright @ 2021 (Springer).

Even pathways like hydrogen production via splitting of water using electricity have gained a widespread attraction among the scientific community (Figure 2) (Wang et al., 2021a). Renewable resources or non-renewable resources are used as feedstock during the electricity driven water splitting (Rozzi et al., 2020). During this reaction, there are two major half-cell reactions taking place: one in which reduction of water occurs at the cathode end to give hydrogen and at the other end oxygen evolution reaction occurs that gives oxygen as the product (Wang et al., 2021a). This reaction occurs in the presence of catalysts like metal based or non-metal based electrocatalysts (Wang et al., 2021a). However, when carbon-based catalysts are used during the reaction, carbon-based byproducts are sometimes formed during this process. The thermocatalytic methane decomposition (TMD) is a promising alternative to the existing methods for carbon dioxide free hydrogen production (Harun et al., 2020). Since the process does not include carbon-based products, steps involved in the elimination of carbon oxides are not required. Furthermore, because of the formation of carbon-free products, there is a significant lowering of greenhouse gas production in contrast to the traditional methods (Ashok et al., 2008a). It has also been reported that hydrogen production through this method reduces CO_x_ emission by about 27% and can also reduce the global climatic effects (Weger et al., 2017; Wang et al., 2021a). The decomposition of methane requires a large amount of energy, almost equivalent to its formation. Being functional at very high temperatures, exceeding 1,300°C, and therefore, the usage of catalysts becomes a necessity to reduce the temperature at which decomposition of methane can easily occur. The present studies have been focused on the development of catalysts that can provide maximum conversion of methane to hydrogen, enhanced activity and stability, and also exhibit low catalytic deactivation (Bockris, 2013; Eljack and Kazi, 2021).

Some of the most commonly used metal catalysts include Ni, Fe, Cu, Co, Pt, Pd, among others. These catalysts are often used alongside a support structure like silica, Mg, Zr, Ti and carbon structures to increase the overall surface area of the catalyst. This is important to provide a larger area to function as catalytic active sites during the reaction (Bockris, 2013; Eljack and Kazi, 2021). Among these catalysts, Ni has gained widespread attention for TMD for hydrogen production, because this element can produce more yield of hydrogen per unit mass of the substrate (Bockris, 2013; Eljack and Kazi, 2021). Moreover, Ni catalysts help form filamentous carbon structures as byproducts that have applications in the nanotechnology field and material sciences (Srilatha et al., 2017). Yet, metal catalysts are highly susceptible to sulfur poisoning and carbidization and therefore are easily deactivated. Dufour and colleagues have performed several studies on TMD and its applications in hydrogen production (Dufour et al., 2009; Dufour et al., 2012). Through their studies, the authors showed that TMD is a better approach towards hydrogen production because of its eco-friendly nature and could occasionally allow for an autocatalytic activity for a limited time (Serrano et al., 2013; Wang et al., 2021a). TMD has been suggested as a solution for the increasing demand for hydrogen in industries and its use in electricity production (Parkinson et al., 2018). Its economic reliability is dependent on the carbon by-product’s value in the market (Keipi et al., 2016). Several kinds of TMD reactors have been explored for the TMD process like the plasma-assisted reactor, molten metal reactor. Besides, many heating points have to be considered to carry out the complete TMD process (Heidenreich et al., 2016; Keipi et al., 2016).

Herein, we provided a detailed overview of hydrogen production via the TMD process. We have discussed thermodynamics and the different methods of hydrogen production. These include, but are not limited to steam methane reforming, partial oxidation of methane, and autothermal reforming. We further discussed the production of hydrogen from renewable sources. These included direct biomass gasification, thermochemical water splitting, methane pyrolysis, aqueous phase reforming, and coal gasification. We have also discussed catalytic deactivation and catalyst regeneration in this review. We finally concluded this article by discussing the future perspectives of the TMD process.

## Thermodynamics

During the TMD reaction, methane molecules are decomposed to give hydrogen (gas) and carbon (solid). That is,

$${\text{CH}}_{4}\to {\;2\text{H}}_{2}\;+\text{ C }\Delta {{\text{H}}^{\text{o}}}_{\;}=\;74\text{.}52\text{ kJ/mole}$$

This reaction is that of TMD via methane pyrolysis and can take place without the requirements of any catalysts (Timmerberg et al., 2020). However, the energy required by the reaction in terms of heat, will be very high (more than 1500 K) (Xiao and Varma, 2018). This data implies that the existence of other catalysts that can influence TMD. Therefore, for TMD reactions at lower temperatures, the requirement of catalysts becomes an utmost need (Gmehling et al., 2019). Researchers have reported that gaseous carbon produced is adsorbed on the catalyst and is diffused into it. This occurs due to diffusion driving force (Baker et al., 1972), which is dependent on concentration or temperature changes. The equilibrium constant of the reaction, K_eq_, is dependent on the kind of catalyst used can be written as (Ibrahim et al., 2015),

$$\frac{{\text{K}}_{\text{eq}}\;=\;{\left({\text{PH}}_{2}^{2}\right)}_{\text{eq}}{\text{C}}^{\text{s}}}{{\left({\text{PCH}}_{4}\right)}_{\text{eq}}}$$

Here, C^s^ is the solubility of carbon in its active phase, while P represents the partial pressures of H_2_ and CH_4_. Also, K_eq_ signifies the equilibrium of the gaseous phase of a carbon mixture having a metal component. The dissolution of filamentous carbon depends on the K_eq_ of carbon, which influences the gaseous phase of the threshold while coking (Ibrahim et al., 2015). That is,

$$\frac{{\text{K}}_{\text{eq }}{=\text{ PH}}_{2}^{2}}{{\text{PCH}}_{4}}$$

The Gibbs energy of TMD also changes with temperature (Villacampa et al., 2003) accordingly:

$$\Delta {\text{G}}^{\text{o}}\left(\text{J/mole}\right)=\;89658\text{.}88\;-102\text{.}27\text{ T }-\text{ 0}{\text{.}00428\text{ T}}^{2}\;-\;2499358{\text{.}99\text{ T}}^{-1}$$

Here, T is the temperature in Kelvin. The above expressions give an estimated value and not precise values because of the involvement of graphite formation. Moreover, the temperature required for the reaction has to be more than 819 K. In a study by Rostrup-Nielsen and colleagues, it was shown that Gibbs energy of TMD (∆G_cd_) is derived by removing the expression of TMD where graphite formation occurs from the original Gibbs free energy (∆G_a_) of methane coking (Rostrup-Nielsen, 1972). That is,

$$\Delta {\text{G}}_{\text{cd }}=\;\Delta {\text{G}}_{\text{a}}\;-\;\Delta {\text{G}}_{\text{o}}$$

Zhang and Smith suggested the threshold constant (K_mf_) for carbon during TMD (Zhang and Smith, 2004; Inaba et al., 2019). This constant defines K_c_, the value at which the rate of deactivation of catalyst nears zero because of carbon synthesis. Hence, stable activity and carbon synthesis in TMD using nickel and cobalt as catalysts is affirmed by considering K_m_ so that,

$${\text{K}}_{\text{mf}}{\text{ < K}}_{\text{c}}{\text{ < K}}_{\text{m}}$$

Ni and Fe catalysts have been rampantly used during the process. Ni based catalysts have shown to have highest activity of 600°C. Morphologies of spent Ni/wood char catalyst at different magnification scales However, methane conversion is found to be thermodynamically limited at this high temperature and therefore large amount of hydrogen cannot be produced in concentrated amounts (Abbas and Wan Daud, 2010). Also, Fe based, though highly stable at higher temperatures, get deactivated easily, and hence have shorter lifetime. So, Chesnokov and colleagues modified the catalysts that included 75%Ni–12%Cu/Al_2_O_3_ with Fe. This showed high functionality at 700–750°C (Chesnokov and Chichkan, 2009) and produced hydrogen products at a high concentration of up to 70 mol%. Similarly Ni-Cu/Al_2_O_3_ catalysts have shown to have considerable advantages over Ni/Al_2_O_3_ catalysts (Abbas and Wan Daud, 2010). At 600–675°C, these catalysts have also shown to possess high metal loading capacity and shows high methane conversions. Likewise, in another study by Wang and coworkers, it was observed that Ni–Cu–MgO catalyst also showed high activity and remained active for longer periods of time at temperature ranged between 665 and 725°C. The authors also noted that this catalysts could produce large amounts of concentrated hydrogen that is devoid of carbon products (Wang and Baker, 2004).

## Methods of Hydrogen Production

**FIGURE 3:**
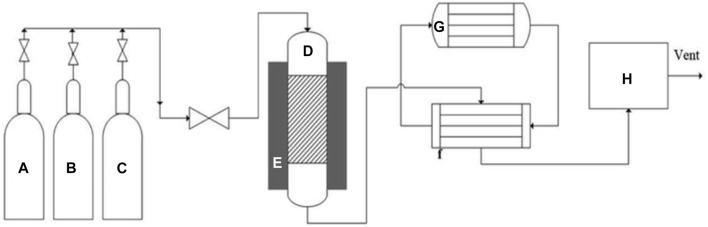
Schematic of experimental set-up for methane decomposition. **(A)** Nitrogen gas cylinder, **(B)** hydrogen gas cylinder, **(C)** methane gas cylinder, **(D)** fixed bed reactor, **(E)** electric heater, **(F)** heat exchanger, **(G)** chiller, **(H)** micro-GC. Adapted from reference Harun et al. (2020) Copyright @ 2020 (Royal Society of Chemistry).

Currently, hydrogen production surpasses one billion m^3^/day. Of this, 48% of this amount is derived via natural gas, oil contributes to 18%, and over 4% comes from water-splitting electrolysis (Nikolaidis and Poullikkas, 2017; Hallenbeck et al., 2019). As discussed previously, hydrogen is produced via several methodologies like TMD, catalytic oxidation, and steam gasification (El-Shafie et al., 2019; Harun et al., 2020) (Figure 3). TMD use many catalysts that may range from metal-based catalysts, noble-metal based catalysts, non-supported catalysts, among others. During the process, a fixed bed reactor is often used (Harun et al., 2020) that contains operating systems for monitoring temperature within the system and the flow rates of the feedstock used.

### Hydrogen Production From Fossil Fuels

**FIGURE 4:**
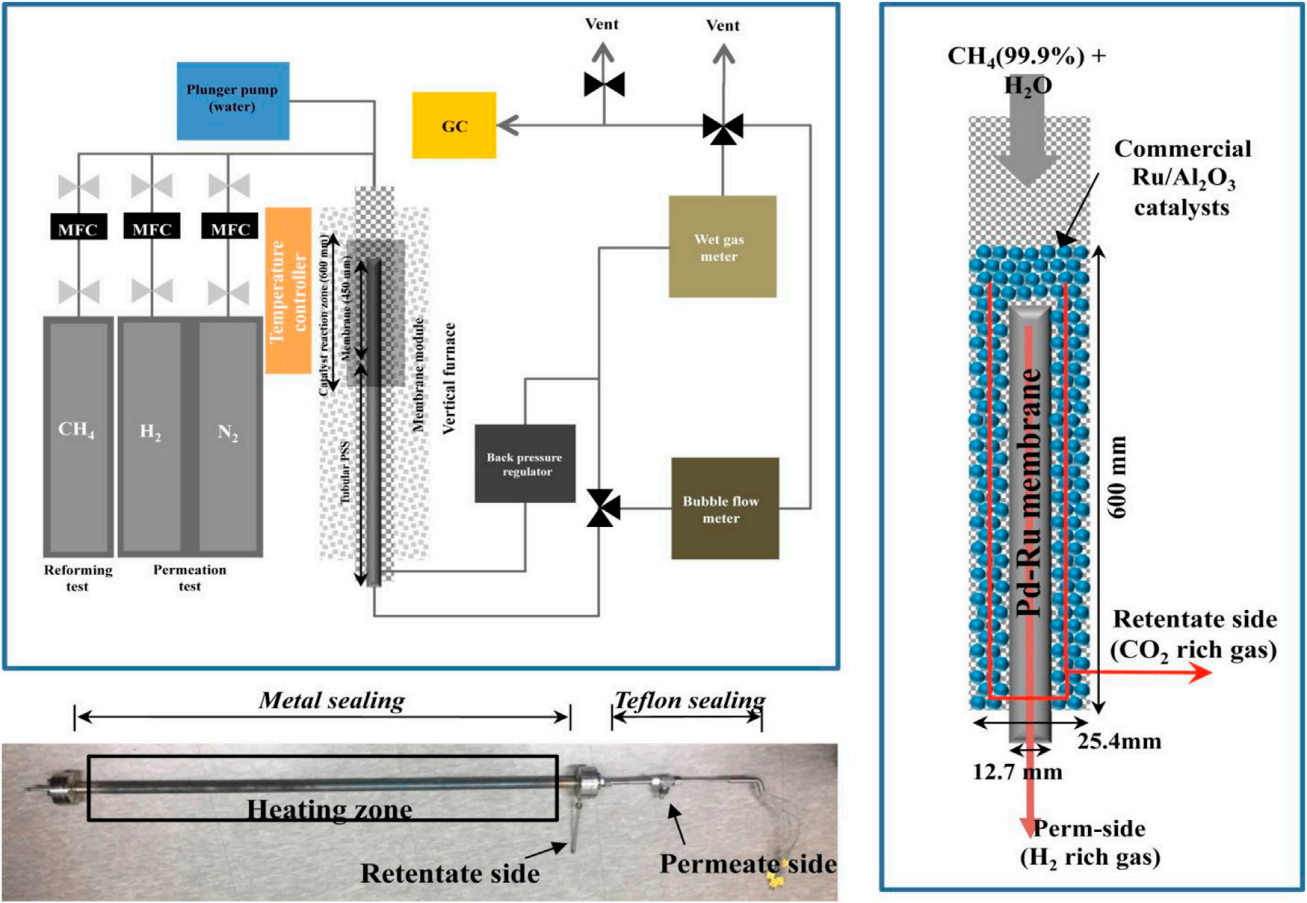
Schematic of the membrane reactor and test setup for steam methane reforming. Adapted from reference Kim et al. (2018) Copyright @ 2018 (Elsevier).

Because of the dominance of fossil fuels, hydrogen is derived mostly from them (Kayfeci et al., 2019). The main processes involved in hydrogen production include catalytic steam reforming of light hydrocarbons, partial oxidation of heavy hydrocarbons, coal gasification, and methane decarburization. The most common method used worldwide is the steam reforming of natural gas (Kim et al., 2018). As shown in the Figure 4, the set up required during steam reforming reaction is quite simple and contains a furnace, a temperature modulator, membrane module, flow and gas meters (Kim et al., 2018). This method is far more environment-friendly and highly efficient (∼70–80%) (Grigoriev et al., 2006; Shiva Kumar and Himabindu, 2019) than other methods that produce hydrogen through fossil fuels (Abe et al., 2019).

#### Steam Methane Reforming

**FIGURE 5:**
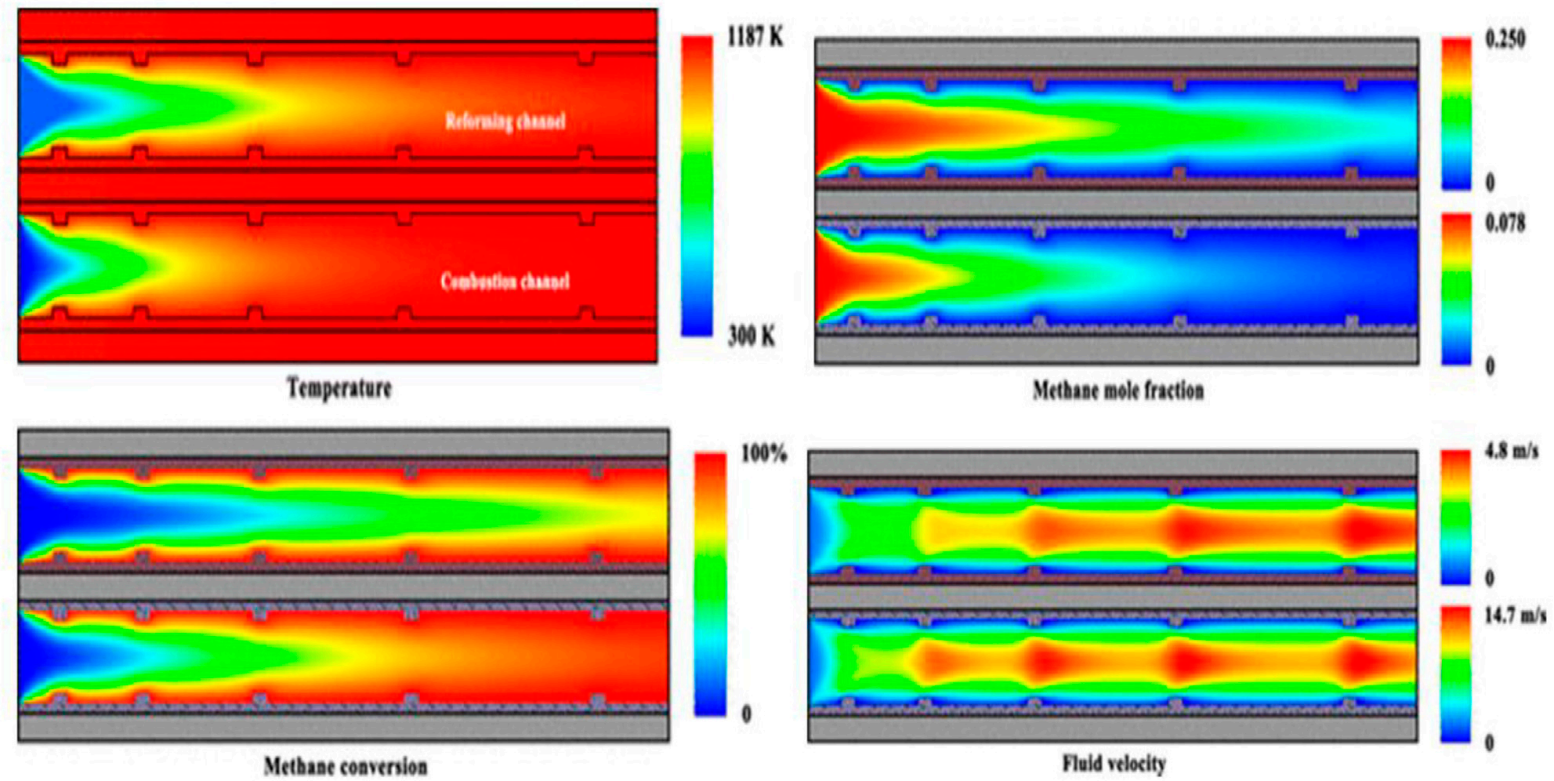
Contour plots of temperature, methane concentration, conversion, and fluid velocity for the reactor. The velocity of the combustible fluid inlet flow is 3.0 m/s. The velocity of the process fluid inlet flow is 2.0 m/s. The steam-to-carbon ratio is 3.0, and the equivalence ratio of the combustible mixture at the inlets is 0.8. The thermal conductivity of the material of the dividing wall is 200 W/(m·K). Adapted from reference Chen et al. (2019b) Copyright @ 2019 (American Chemical Society).

Steam methane reforming (SMR) is usually used for industrial purposes to produce hydrogen from methane sources, like from fossil fuels (coal, natural gas, etc.). The process is carried out at a very high temperature of 700–1,000°C, and pressure ranged between 3 and 25 bar (Chen et al., 2019b; Chen et al., 2020a) (Figure 5). When this reaction is carried out along with the water-gas shift (WGS) reaction, a further reaction occurs between CO and steam to create additional H_2_ and CO_2_ (Ertl et al., 2008).

During the methane steam reforming reaction, hydrogen is formed, further utilized to produce ammonia, methanol, and other hydrocarbons. Two reactions occur during the SMR process.

$${\text{CH}}_{4}{\;+\text{ H}}_{2}\text{O }⇌{\text{ CO }+\;3\text{H}}_{2}\;\Delta {\text{H}}_{298}^{\text{0}}=\;205{\text{.}8\text{ kJmol}}^{-1}$$

$${\text{CO }+\text{ H}}_{2}\text{O}⇌{\text{ CO}}_{2}{\;+\text{ H}}_{2}\;\Delta {\text{H}}_{298}^{0}{=-41\text{ kJmol}}^{-1}$$

The mechanism of the reaction determines the activity of the catalyst during the reaction. When the deposition of carbon occurs during the dehydrogenation step, it is a competitive reaction and is the rate-determining step. Many studies indicate that, during the reaction, CO adsorbs on the active sites of the catalysts, forming CO_2_ (Kramer et al., 2018; Niu et al., 2020). Moreover, there is a considerable increase in the temperature of the reaction during the deposition of carbon and also a formation of a surplus amount of steam during the reaction process.

#### Partial Oxidation of Methane

**FIGURE 6:**
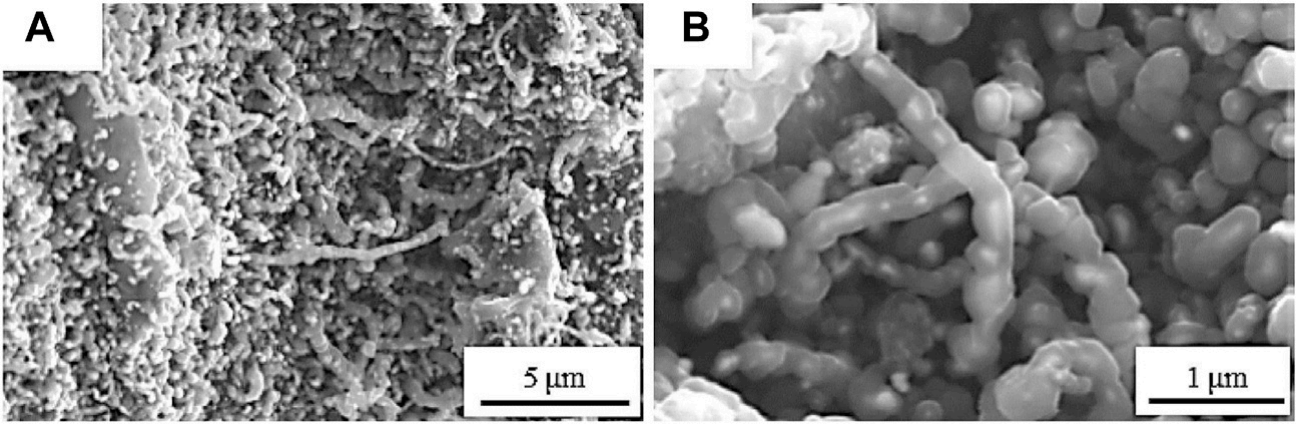
Morphologies of spent Ni/wood char catalyst at different magnification scales. Adapted with permission from reference Hasnan et al. (2020b), copyright@2020 (Springer).

POM is an exothermic reaction that usually gives rise to syngas and other oxygenated compounds like formaldehyde, ethylene, and many hydrocarbons (Chao et al., 2008). Of all the catalysts, Ni is the most popularly used in this reaction because it is cheaply available (Daneshmand-Jahromi et al., 2017; Ha et al., 2020). The morphologies of the spent nickel/wood char catalyst is shown in Figure 6.

POM reaction is a far more efficient hydrogen production technique because of the lack of big reactors and mega superheated steam. This is because of the exothermic nature of the reaction (Ghoneim et al., 2016). Moreover, the hydrogen to CO ratio in this reaction allows for methanol usage and Fischer Tropsch synthesis without any further changes. The stoichiometry of the reaction is as follows:

$$CH_{4}+1/2\;O_{2}⇆\mathrm{CO}+2H_{2}$$

However, this reaction poses a few challenges, such as the inability to control the reaction’s selectivity during complete combustion.

#### Autothermal Reforming

**FIGURE 7:**
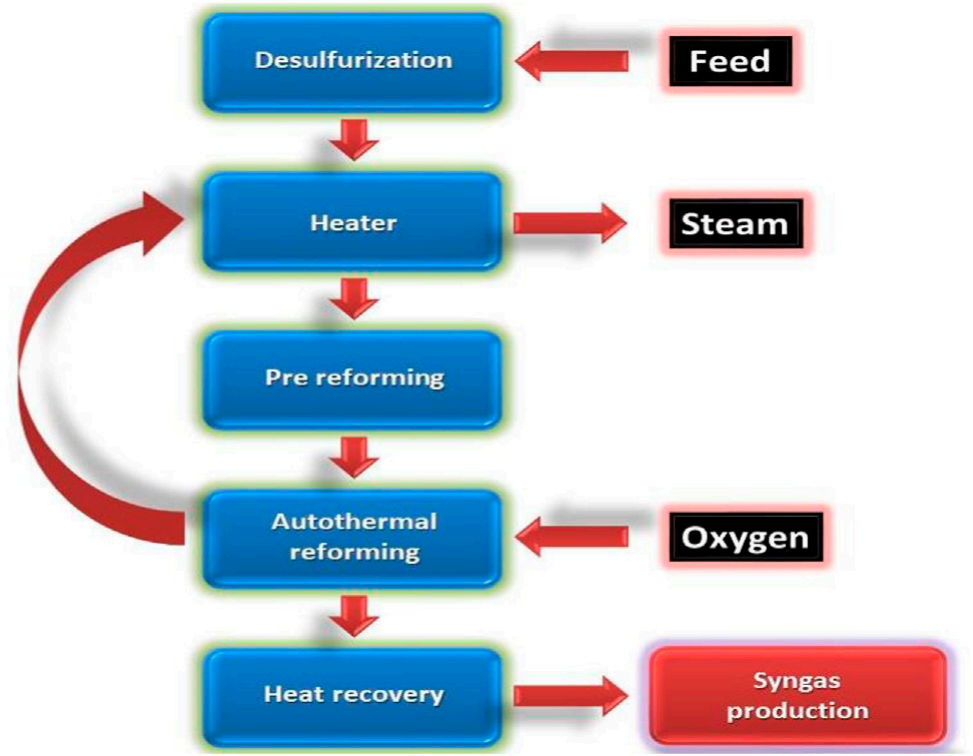
Contrast between autothermal reforming and syngas production.

In the autothermal reforming method (also called the oxidative steam reforming method), oxygen and carbon dioxide react with methane to produce hydrogen. Owing to the oxidation steps in this reaction, it is exothermic. In contrast to the steam methane reforming method, this reaction involves the usage of oxygen. Figure 7 shows this contrast between the two reactions. This main advantage of this method is that, the hydrogen and CO ratio can be monitored (Patil et al., 2007; Carapellucci and Giordano, 2020). This is useful because this process can produce biofuels that need an equal proportion of hydrogen and CO.

The efficiency of ATR depends on hydrogen production. Owing to thermodynamic restriction, low amounts of hydrogen production may potentially hinder the reaction progression. Another limitation of the process is the involvement of air. If air is involved in the reaction, then additional steps are required to separate the products. This, in turn, leads to a higher cost of production (Pinton et al., 2017). The general reaction during autothermal reforming is:

$${\text{C}}_{\text{x}}{\text{H}}_{\text{y}}{\text{O}}_{\text{z}}\;+\;[\text{2x }-\left(\text{z}+1\right){]\text{ H}}_{2}{\text{O }+1/2\text{ ½ O}}_{2}\to {\text{xCO}}_{2}\;+\;\frac{2[\text{2x }-\left(\text{z}+1\right)]}{2}{\;+\text{ yH}}_{2}$$

For instance, during ATR of ethanol, combinational reaction is carried out simultaneously (Chen et al., 2009). These include partial oxidation follow by steam reforming. The following reactions occur:

$${\text{C}}_{2}{\text{H}}_{5}{\text{OH }+1/2\text{ ½ O}}_{2}\to {2\text{CO }+\;3\text{H}}_{2}$$

$${\text{C}}_{2}{\text{H}}_{5}{\text{OH }+1/2\text{ ½ O}}_{2}{\;+\;2\text{H}}_{2}\text{O }\to {\;2\text{CO}}_{2}{\;+\;5\text{H}}_{2}$$

These reactions occur in between 500 and 800°C (Chen et al., 2009).

### Hydrogen Production From Renewable Sources

Despite the rampant production of hydrogen from fossil fuels, those methodologies pose serious concerns, especially environment-related issues and higher production costs. Being a greenhouse gas, it poses a lot of concern towards environmental sustainability. Hence, hydrogen production’s better alternative is from renewable resources like geothermal sources, biomass, wind energy, etc., (Avcıoğlu et al., 2019). There are methods available that permit the chemical conversion of renewable resources to give rise to hydrogen. Such strategies include biomass gasification, steam reforming, among others. The hydrogen produced is used in metallurgical purposes, electronics development, and various other chemical applications (Srilatha et al., 2017).

#### Direct Biomass Gasification

The main reactions in DBG are endothermic in nature and involves partial oxidation processes (Kumar et al., 2017; Salam et al., 2018). The energy for the reaction is provided by the oxidation of biomass via autothermal reaction. There are four main steps involved in this process: oxidation, drying, pyrolysis, and reduction. The general stoichiometry of the main reaction (Molino et al., 2016) that occurs during the gasification process is:

$${\text{nCO }+\;2\text{nH}}_{2}\;\to {\text{ C}}_{\text{n}}{\text{H}}_{2\text{n}}\;+\text{ OH }+\;\left(\text{n}-1\right){\text{H}}_{2}\text{O}$$

**FIGURE 8:**
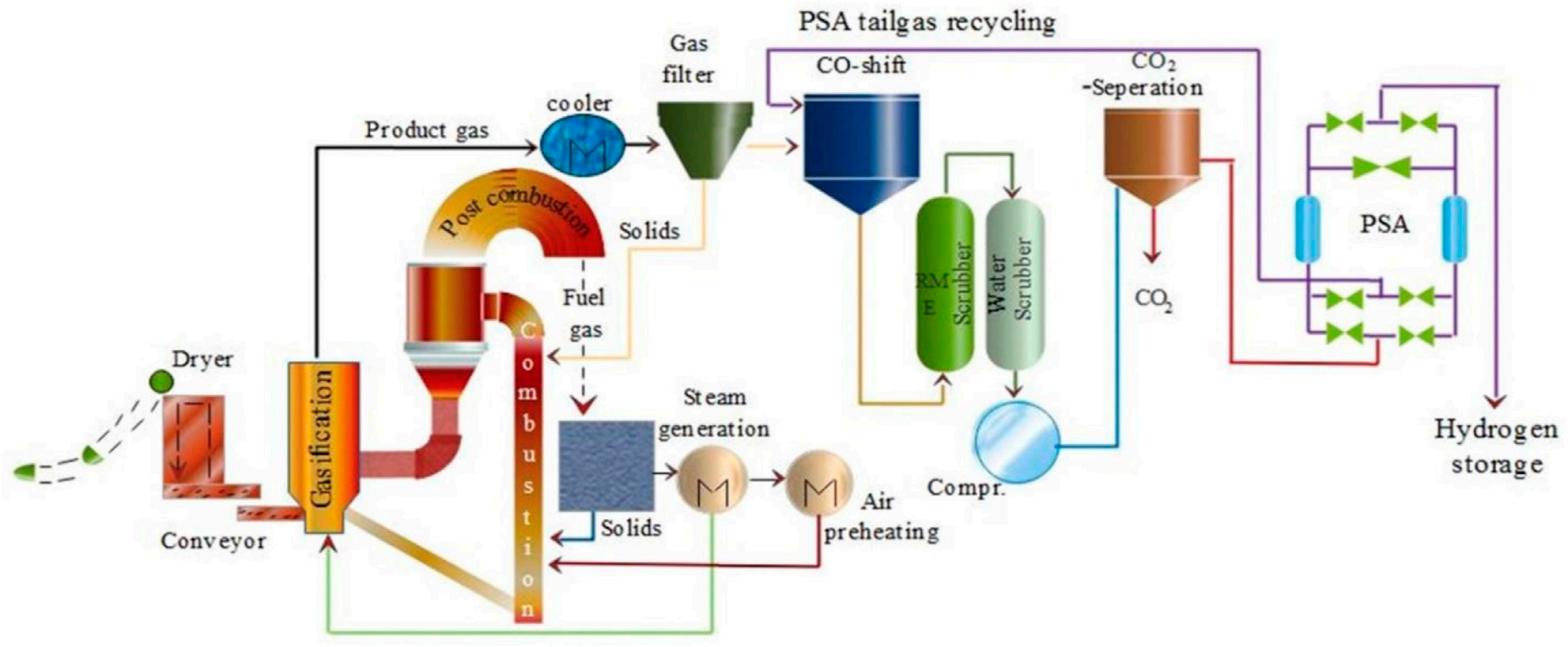
Process flow diagram of biomass to hydrogen. Adapted from reference Salam et al. (2018) Copyright @ 2018 (Elsevier).

A flow diagram depicting the hydrogen production via biomass gasification is shown in Figure 8 (Salam et al., 2018). The set up requires limited air or steam that functions as gasifying agents. Scrubbers like RME and water scrubbers are used to clean and dry during the process. To remove any carbon based products formed during the process, a special membrane separation unit is present. Then a pressure swing adsorption (PSA) channel is present that gives concentrated hydrogen products.

#### Thermochemical Water Splitting

**FIGURE 9:**
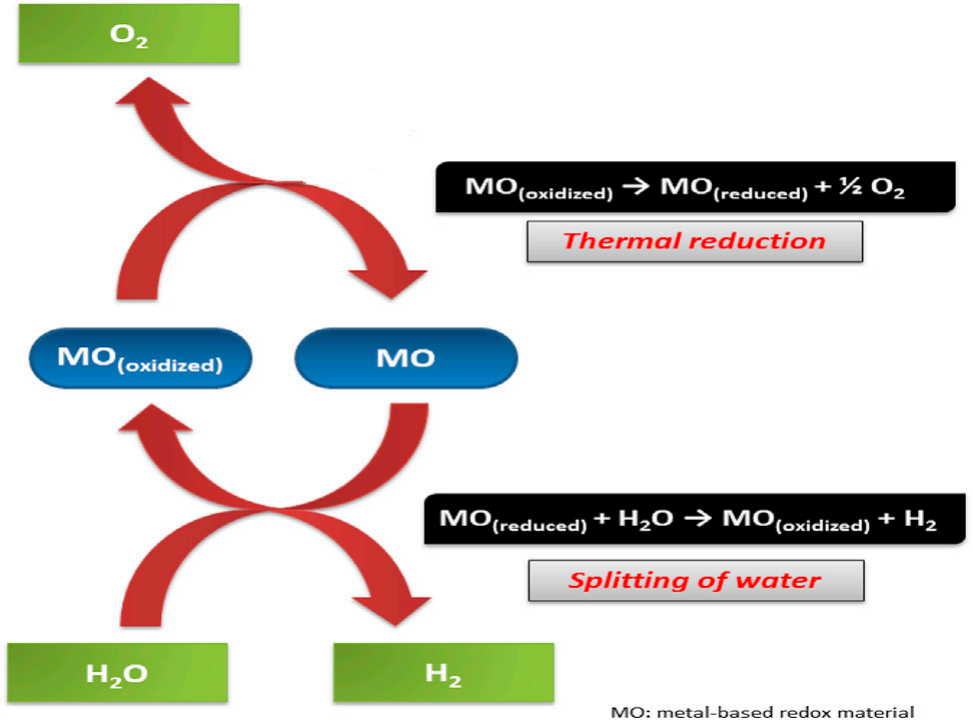
Thermocatalytic water splitting reaction that produces hydrogen molecules.

The TWS process involves a direct transformation of thermal energy to hydrogen. Hydrogen is produced in abundance and hence serves as an energy carrier in various techniques (Safari and Dincer, 2020). The reaction is carried out at very high temperatures – above a thousand degrees. The reaction occurs by simultaneously carrying two chemical reactions. First, elevated temperature endothermic process and lowered temperature exothermic process. This reaction’s net result is a thermochemical water-splitting process that gives an abundance of free energy post-reaction (Budama et al., 2021). In the thermo catalytic reaction (Figure 9), the metal catalyst (usually metal oxide) undergoes reduction during the first endothermic step. This step is also called the activation step. In this step, oxygen is given out, further reacting with water in the hydrolytic step. The second step is an exothermic reaction to finally give out hydrogen and oxide – which is then recycled in the previous step (Safari and Dincer, 2020).

The splitting of hydrogen and oxygen is an uphill reaction. A significant positive change in Gibbs free energy is observed during this reaction. This means ΔG⁰ = 237 kJ/mol is produced during the reaction. Hence, because of the high energy of the reaction, it is also called artificial photosynthesis.

$${\text{H}}_{2}\text{O }\left(\text{l}\right)\;\to {\text{ H}}_{2}\left(\text{g}\right){+1/2\text{ ½ O}}_{2}\left(\text{g}\right)$$

The energy for the reaction is made available for splitting H-O-H bonds is provided by electrical, electromagnetic, or thermal sources. The process is carried out by transferring electrical current into the water. Here, the chemical energy is produced from electrical energy (Landman et al., 2017). At the anode, water disintegrates into five oxygen and protons. While at the cathode, hydrogen is produced.

#### Methane Pyrolysis

When hydrogen is produced by decomposing methane, the reaction method is referred to as pyrolysis or MP, or methane cracking. In this process, methane gets split into its component elements that are H_2_ and solid C. The combustion of carbon does not occur within this process. In this method, hydrogen is produced inexpensively when compared to steam methane reforming. Moreover, CO_2_ capture and storage (CCS) also occur during this process (Timmerberg et al., 2020; Leal Pérez et al., 2021). The reason for the cheap reaction process could be the production of solid carbon. This solid carbon is precious and hence compensates for the costs of the entire process. The process of methane pyrolysis undergoes the chemical splitting of methane. The resultant compounds include hydrogen and hydrocarbons, along with solid carbon molecules (Timmerberg et al., 2020).

$${\text{CH}}_{4}\left(\text{g}\right)\to \text{C}\left(\text{s}\right){+2\text{H}}_{2}\left(\text{g}\right)\Delta {\text{H}}_{298\text{K}}\;=\;74\text{.}52\text{ kJ/mol}$$

The process is endothermic and derives energy from various sources. Since oxygen is not involved in the reaction, no carbon dioxide or its derivatives are produced. Hence there are no further separation processes required during the reaction and, therefore, are less complicated. However, sometimes the hydrogen produced may need to undergo further processing to remove any mixture’s impurities. When only hydrogen is expected to be the sole product, then the reaction’s total efficiency remains 59%, and the rest gets trapped in the solid carbon produced.

#### Aqueous Phase Reforming

APR also synthesizes hydrogen from biomass, including sugar, glycerol, and oxygenated compounds. This reforming is carried out in a liquid state of the reactants. The major plus point of this reaction is that hydrogen is produced without the evaporation of water. This, therefore, saves a lot of energy during the process.

APR was first developed by Davda and colleagues (Davda et al., 2005). In the first step, water is added to the reactant biomass. This gets separated into aqueous and organic phases. The aqueous phase is then transported to another hydrogenation area where hydrogenation of the aqueous phase occurs. In the hydrogenation step, hydrogen is synthesized via APR (Vispute and Huber, 2009). Glucose is also used as a reactant during the APR reaction for hydrogenation. Glucose is an ideal candidate because of the reduction in the number of reaction steps (Fasolini et al., 2019): glucose is synthesized during the aqueous stream. APR of glucose can be shown as:

$${\text{C}}_{6}{\text{H}}_{12}{\text{O}}_{6\;}{+\;6\text{H}}_{2}\text{O}\to {12\text{H}}_{2}{\;+\;6\text{CO}}_{2}$$

In general, the reaction can be expressed as:

$${\text{C}}_{\text{n}}{\text{H}}_{2\text{y}}{\text{O}}_{\text{n }}\to {\text{ nCO }+\text{ yH}}_{2}$$

This reaction, however, is oversimplified and is not a direct reaction. There are several intermediates formed during the reaction. Hydrogenation of CO/CO_2_ results in the formation of alkanes. On the other hand, dehydration gives ketones and aldehydes.

#### Coal Gasification

This technology of CG is a recent development**.** This process has many advantages, for instance, high solubility, transfer of mass, and quick heating of coal (Stiegel and Ramezan, 2006). Moreover, low carbon deposition on the catalysts occurs, and the gasification process is highly efficient. Coal is changed to abundant hydrogen gas at lower temperatures in contrast to the conventional gasification processes. Furthermore, compounds having an abundance of nitrogen and sulfur are wholly converted in the aqueous phase (Williams et al., 2018). This, therefore, reduces the overall pollution of the reaction.

During coal gasification, coal undergoes a reaction with steam and air (containing oxygen). During the reaction, organic material reacts in the presence of a catalyst(s) to give carbon-monoxide, hydrogen, methane, and small concentrations of other gases containing nitrogen and sulfur. Ash (char) is also produced as a product (Widjaya et al., 2018). During volatilization, the general stoichiometry of the reaction is:

$${4\text{C}}_{\text{n}}{\text{H}}_{\text{m}}\;\to {\text{mCH}}_{4}\;+\;\left(4\text{n }-\text{ m}\right)\text{C}$$

During the char gasification step, the following reaction occurs:

$${\text{C }+\text{ H}}_{2}\text{O }\to {\text{ CO }+\text{ H}}_{2}$$

$${\text{C }+\text{ CO}}_{2}\to 2\text{CO}$$

$${\text{C }+\;2\text{H}}_{2}\to {\text{CH}}_{4}$$

The gas-phase reactions include:

$${\text{CO }+\text{ \_O}}_{2}\to {\text{ CO}}_{2}$$

$${\text{H}}_{2}{\;+\text{ \_O}}_{2}\;\to {\text{ H}}_{2}\text{O}$$

$${\text{H}}_{2}\text{O }+\text{ CO}\to {\text{ H}}_{2}{\;+\text{ CO}}_{2}$$

$${\text{CO }+\;3\text{H}}_{2}\to {\text{CH}}_{4}{\;+\text{ H}}_{2}\text{O}$$

The rates at which the complexes are formed and removed; and the number of active sites occupied on the catalyst’s surface determine every individual step’s rate and order.

## Catalysts

Catalysts are most commonly synthesized by the process of impregnation owing to its simplicity at laboratory and industrial levels. The process involves impregnation of porous support substances in the presence of metal oxide solutions (Van Dillen et al., 2003). Then the solvent is evaporated giving the desired catalyst. However, there is a limited reaction between the metal precursor and support, and also the solvent evaporates over the support particles causing a capillary flow of the solution. Thus the formation of egg shell catalysts occurs that do not possess good active phase dispersion. In the ground breaking work done by Kotter and Riekert, it was discovered that with an increase in viscosity of impregnating solution, the outward flow of the solution reduces that causes a better activity on supporting elements (Van Dillen et al., 2003). Other processes involved in catalyst synthesis show either extremely high or extremely low drying rates with moderate quality type of products (Jackson, 1995).

### Metal Catalysts

Metal catalysts like Ni/Al_2_O_3_ are better preferred during SMR reactions because of their inexpensive nature (Siew et al., 2014). The most extensively studied metal catalysts for this process are from the VIII group of the periodic table. Their properties, like the size effect (Vogt et al., 2020), support effect (Wang et al., 2018), promoter effects (Cao et al., 2020), and coordination states (Rogers et al., 2016), have also been well researched. Even the external conditions of methane reforming like the temperature, pressure, and kind of reactor used, are considered while using the metal catalysts.

Transitions metals like Fe and Ni that have semi-filled d-orbitals are also used in SMR reactions because of their enhanced stability and activity (Syed Muhammad et al., 2018; Inaba et al., 2019). Ni catalysts are far more convenient for TMD via SMR reactions when carried out at 500–600°C. However, this temperature range is lower than the conditions needed to achieve an equilibrium state because Ni deactivates at higher temperature conditions. On the contrary, Fe functions efficiently at increased temperatures and is comparatively less expensive (Frontera et al., 2017). The most commonly used metal catalysts are the transition metal catalysts like Ni, Co, and Fe. Because of the enhanced turnover rates and being cheaply available, Ni catalysts are better preferred. Studies have also shown that, in the presence of elements like rhodium, Ni gets easily reduced, which increases the methane conversion, thereby increasing the total hydrogen production (Kim et al., 2015). Table 1 shows the data related to a few recent reports about hydrogen production via POM method using Ni-based catalysts supported on various catalysts.

**TABLE 1:** Various Ni-based metal catalysts used for hydrogen production via POM method.

Metal	Support	Methane conversion (%)	Catalyst preparation method	H_2_/CO	References
Rh-Ni	Al_2_O_3_	∼90	Wet impregnation and solid-state reaction	∼2	Alvarez-Galvan et al. (2019)
Rh-Ni	CeO_2_	∼80	Wet impregnation and solid-state reaction	∼2	Alvarez-Galvan et al. (2019)
Rh-Ni	La_2_O_3_	∼50	Wet impregnation and solid-state reaction	∼2	Alvarez-Galvan et al. (2019)
Rh-Ni	MgO	∼50	Wet impregnation and solid-state reaction	∼2	Alvarez-Galvan et al. (2019)
Rh-Ni	ZrO_2_	∼50	Wet impregnation and solid-state reaction	∼2	Alvarez-Galvan et al. (2019)
Ni	Al_2_O_3_ and ZrO_2_	90	Wet-impregnation method	2	Fakeeha et al. (2020)

Like the other processes, the most widely used metal catalyst is based on the transition element, Ni (Farsi and Mansouri, 2016), because of its cheap availability and reaction efficiency. The Ni available in the catalyst system usually remains below 20% due to its highest activity observed at that concentration (Haynes and Shekhawat, 2011; Scapinello et al., 2017). This could occur because of a decrease in Ni dispersion with increasing Ni amount. Studies have reported that catalysts prepared via microwave assistance impregnation methods show enhanced activity (Hosseini et al., 2017). Moreover, microwave radiations enhance the rate at which Ni gets deposited on the catalyst’s surface during their preparation. The efficiency of such a catalyst and their influence in methane reforming reactions was observed in the studies carried out by Roh and colleagues (HS et al., 2002). The authors explained this behavior by the catalysts due to the high interaction with the catalysts’ active phase. Table 2 shows the data related to a few recent reports about hydrogen production via autothermal reforming reactions.

**TABLE 2:** Various Ni-based metal catalysts used for hydrogen production via autothermal reforming of methane.

Metal	Support	Methane conversion (%)	H_2_/CH_4_	References
Ni	SiO_2_Al_2_O_3_	∼100%	1	Ali et al. (2016)
Ni	MgAl_2_O_4_	∼75%	5	Katheria et al. (2016)
Ni	Ni_2_Al_2_O_5_	82%	2.4	Rogers et al. (2016)
Ni	ZnLaAlO_4_	∼72%	3	Khani et al. (2016)
Pt	ZnLaAlO_4_	∼88%	3	Khani et al. (2016)
Ru	ZnLaAlO_4_	∼72%	3	Khani et al. (2016)
Ni	γ-Al2O3	∼98%	3	Khani et al. (2016)

Many catalysts like the ones based on nickel, zeolites, alumina, platinum, ruthenium, and others based on alkali metals have been studied (Okolie et al., 2019). Ni-based catalysts have shown high-end results during the gasification process because of their ability to allow reforming reactions (Nikolaidis and Poullikkas, 2017). Studies have revealed that alumina silicate-based catalysts are far more effective than Ni-based catalysts during the gasification process (Arregi et al., 2018). Nickel-based catalysts are often used when hydrogen is the only product needed. Its activity is dependent on the presence or absence of the support material and other additives (Kannaiyan et al., 2016). Table 3 shows the data related to a few recent reports about hydrogen production via direct biomass gasification method using Ni-based catalysts supported on various catalysts.

**TABLE 3:** Various Ni-based metal catalysts used for hydrogen production via methane reforming reactions.

Metal	Support	Methane conversion (%)	H_2_/CO	References
Ni	Al_2_O_3_	86.17	1.49	Peng et al. (2017)
Ni	CeO_2_/Al_2_O_3_	93.65	1.84	Peng et al. (2017)
Ni	Ru/γ-Al_2_O_3_	80	1.5	Calzada Hernandez et al. (2020)
Ni	MCM-41	∼100	3.85	Akubo et al. (2020)

Metal catalysts show enhanced activity and high chemical and mechanical efficiency and stability during the water-splitting reaction. Metals like Ni, Co, Pt, and others are very commonly used during the reaction (Konsolakis et al., 2016). Among these, Ni catalysts have shown the highest effectiveness for hydrogen production because of the resultant large yield during the reaction (Saikia et al., 2020; Wang et al., 2021b). However, metal poisoning by sulfur and carbon deposition on their actives sites results in metal deactivation. Hence, even carbon-based catalysts are used to overcome the shortcomings of metal-based catalysts (Dufour et al., 2010; Harun et al., 2020).

Most metal catalysts that are used in this reaction get deactivated on carbon deactivation on their surface. The separation of the coke deposited on the surface can be a cumbersome process. We have discussed catalyst reactivation in the following sections. Because of the additional reactivation process for catalysts, sometimes no catalysts are preferred. Moreover, metal-based catalysts are also promising entities for the process because they still show better stability and efficiency than carbon-based catalysts.

Among the commonly used metal catalysts, nickel has shown high conversion rates even at 500°C temperatures. While, without a catalyst, the reaction proceeds at a higher temperature of 700°C (Brandon and Kurban, 2017). Apart from Ni, even Fe-based catalysts have shown to be inexpensive and widely used for the industrial production of hydrogen via methane pyrolysis (Qian et al., 2020). APR reactions that produce hydrogen have been studied for various catalysts, including iron, nickel, palladium, rhodium, iridium, etc. These elements have been noted to show high activity as catalysts during aqueous phase reforming for hydrogen production. Among these, platinum, palladium, and nickel-tin alloys have displayed enhanced activity from hydrogen production. It has been noted that when and if the supports demonstrate basic or neutral nature, then the selectivity during hydrogen production and its selectivity increases. Table 4 shows the data related to a few recent reports about hydrogen production via APR method using Pt-based catalysts supported on various catalysts.

**TABLE 4:** Various Pt-based metal catalysts used for hydrogen production via APR method.

Metal	Support	Hydrogen yield	Conversion (%)	References
Pt	CMK-3	57.5%	79.4	Kim et al. (2012a)
Pt	CMK-9	94.2%	89.2	Kim et al. (2012a)
Pt	CMK-3-MCN-R	71.7%	84.7	Jeong et al. (2014)
Pt	CMK-5-MCN-R	8.6%	88	Jeong et al. (2014)
Pt-Re	CMK-3	19.9 cm^3^/(g_cat_·min)	44.2	Kim et al. (2012b)
Pt-Ni	γ-Al_2_O_3_	—	80	De Vlieger et al. (2012)
Pt-Fe	γ-Al_2_O_3_	48.1 cm^3^/(g_cat_·min)	48.1	Huber et al. (2006)
Pt-Re	CMK-3	36.6 cm^3^/(g_cat_·min)	89.3	Kim et al. (2012b)

As in the previously discussed methodologies for hydrogen production, Ni-based catalysts have the maximum catalytic activity and efficiency. It helps in adjusting the ratio of CO_x_ and hydrogen during the conversion of methane. The highest activity of this catalyst is observed at 780°C (Widjaya et al., 2018). However, as discussed previously, the Ni catalysts get deactivated due to carbon deposits. On the contrary, Ni is relatively cheap and readily available; hence, it is an ideal catalyst and shows optimal activity.

### Metal Supported Catalysts

Metal supported catalysts like catalysts supported by Ni are also preferred in SMR reactions. They are relatively cheaper and available with ease. Moreover, they show enhanced activity. This was observed in the study carried out by Karaismailoglu and colleagues (Karaismailoglu et al., 2019). They used Ni-based catalysts attached to yttria. The authors developed a catalyst via the sol-gel method. They noticed its activity between 390 and 845°C and observed that the maximum amount of coke was formed when the temperature was elevated. Few studies indicate that methane decomposition can occur without the reduction of catalysts (Musamali and Isa, 2018). Such methods are relatively more inexpensive. Moreover, it has been noted that hydrogen production reduces this way. Table 5 shows the data related to a few recent researches about hydrogen production via SMR method using Ni-based catalysts supported on various catalysts.

**TABLE 5:** Various Ni-based metal catalysts used for hydrogen production via methane reforming reactions.

Metal	Support	Methane conversion (%)	H_2_/CO	Ref
Ni-Co	Al_2_O_3_–MgO	79.17	1	Abd Ghani et al. (2018)
Ni	Nd-mesoporous silica	53	0.75	Li et al. (2017)
Magnesium-NiO	Mesoporous zirconia	82	0.94	Al-Fatesh et al. (2020)
Ni	MSC-1	85	∼1	Zhang et al. (2019b)
NiO	MgO–Al_2_O_3_	91	∼1	Chai et al. (2017)
Ni	La@KCC-1	∼96	∼1	Abdulrasheed et al. (2020)
Ni	Ce-ZnAl_2_O_4_	82	1	Movasati et al. (2017)

The most commonly used metal-supported catalysts during the direct biomass gasification process include Ni-based catalysts having K_2_CO_3_ support. Even fundamental catalysts like MgO and CaO are also used during the reaction (Anis and Zainal, 2011). However, these metal-supported catalysts make use of sorbents that are eco-friendly to enhance the catalyst’s overall sensitivity. Limitations also persist in the noble metal-supported catalysts that the ones supported by rhodium (Asadullah et al., 2004). Among the metal catalysts like nickel, cobalt, palladium, iron, and others, none of these shows a very high efficiency on their own during the thermo catalytic water splitting reactions. Hence elements like silica, alumina, magnesia, carbon are used simultaneously along with the core catalysts. These metal support catalysts enhance the total available surface area on the catalyst so that more active sites can be made available to enhance the reaction process (Srilatha et al., 2017).

The second metal support is introduced in a catalyst to increase the life of the catalyst. For instance, when the second metal support is introduced in Ni-based catalysts, the activity and its stability have shown to be exponentially increased (Sánchez‐Bastardo et al., 2020). The most popularly used metal-supported catalysts include the ones supported by palladium and copper. Because of the larger surface area, the catalyst deactivation does not occur immediately, and hence the catalysts remain stable for longer durations. The support available for the catalysts allows for equilibrium between the methane dissociation and carbon diffusion rates. Certain metals like palladium and copper enhance the reduction process. Moreover, weak bonds between metals and their supports also improve metals’ reducing capacity; when there are strong bonds between metal and the support in a catalyst, the catalyst’s dispersion and stability increases.

Tao and colleagues studied the effect of nickel-iron-cobalt alloy in APR reaction (Tao et al., 2020). The authors observed that the nickel-iron allowed for better catalytic activity during the reaction than the lone nickel and iron catalysts. Moreover, when cobalt is added to the catalyst, the catalyst’s overall activity was shown to increase. A higher conversion rate of the reactants was observed in aqueous phase reforming, and more hydrogen production was recorded. Moreover, the selectivity of hydrogen was also shown to increase tremendously. This catalyst was shown to undergo reduction at even low temperatures of 300°C. However, the activity was recorded to increase with increasing temperature. Maximum activity was observed at ∼660°C.

Earth metal catalysts have been shown to play a useful role during hydrogen production during coal gasification. Dolomite (CaCO_3_.MgCO_3_) has very high efficiency and does not need to undergo any regeneration after the catalytic process (Widjaya et al., 2018). Other than those, nickel-aluminum catalysts and nickel-olivine catalysts have also shown practical usage during coal gasification of methane for hydrogen production (Mei Wu et al., 2014). Ni-based catalysts, in general, are involved in removing tars and excess methane.

### Non-Supported Catalysts

Studies also indicate that the performance of a given catalyst for TMD depends on the material of carbon used as the support (Yang et al., 2015). Among these, graphene has better activity when compared to unsupported catalysts (Hasnan et al., 2020a). This was seen in the work of Lua and Wang (2013). They studied non supported Ni-based catalysts in TMD and discovered that CH_4_ must be inserted in the reaction mixture at a reduced temperature for an efficient reaction process. Unsupported catalysts like NiCuAl (Suelves et al., 2006) and NiCuMg (Moliner et al., 2008) exhibit high activity and stability. They provide an opportunity to develop metal crystals that allow for the reaction to proceed for prolonged durations. Hence, non-supported catalysts also play a vital role in hydrogen production. Despite being cheaply available, transition metal catalysts like the ones based on Ni are easily deactivated when on stream. This may occur because of frittage, deposition of carbon on the surface, or further reactions with the substrates (Pantaleo et al., 2016). The activity of these catalysts depends on their active forms and the support used during the reaction. The size of the metal particles is an essential feature that determines their intrinsic activity and their deactivation rate. These features are found to be inversely proportional to the size of the metal particles (Kosinov et al., 2019).

When another metal is attached to these metal-based catalysts like Ni-based catalysts, the resultant catalyst’s stability dramatically increases. Metals like rhodium, platinum, palladium, iridium have been used in metal catalysts to improve their overall strength. This has been seen in many studies (Alvarez-Galvan et al., 2019). Of all the metals used to support other metal catalysts, rhodium is the most promising of all (Tanaka et al., 2010). This is because it aids in the non-noble metal to remain a metal by disallowing the excess transfer of hydrogen atoms from noble metal to non-noble metal catalysts (Arandiyan and Li, 2012). As discussed above, Ni catalysts serve as the most popular option for catalysts during hydrogen production. Apart from this, palladium, copper, and other metals are also used as catalysts. However, these metals alone sometimes lead to the deactivation of the catalysts. This happens because of the carbon deposition at the catalysts’ surface.

**FIGURE 10:**
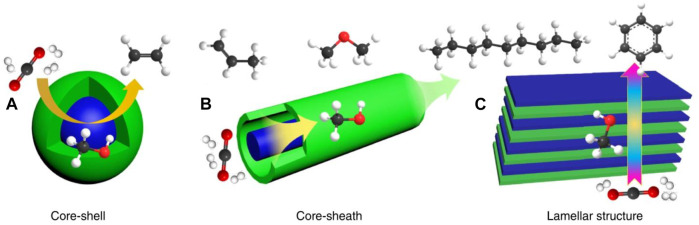
Proposed structures of bifunctional catalysts. Adapted with permission from reference Ye et al. (2019), copyright@2019 (Nature).

This deactivation can be prevented by adding metals like iron, platinum, gold, etc., along with the primary metal catalyst to improve the catalysts’ overall activity and efficiency. This was shown in a study done by Dantas and colleagues (Dantas et al., 2010). The scientists reported that the catalyst supported on silver allowed for the increased conversion of methane and accounted for the stability of the catalyst function and activity. In another study, Kaori Yoshida and coworkers discovered that Ni catalysts supported on palladium give the most promising results during hydrogen production (Yoshida et al., 2009). Such metal-supported catalysts have shown to provide more hydrogen/CO ratio when compared to unsupported catalysts. The supported catalysts support enhanced catalytic activity, enhanced stability, and larger active surface area for the catalyst to function. Any support gives porosity that gives increased contact with reactants. Moreover, the nature of contact between the support element and the catalyst, and the bond between them determines the reaction rate during hydrogen production. Studies have shown that the addition of Mg, Co, Zn elements increases Ni-based catalysts (Zhu et al., 2011). Also, ZrO_2_ is an unbound or non-supported catalyst that leads to increased efficiency for hydrogen production via an autothermal oxidation reaction (Jeong et al., 2006). In addition to these catalysts, bifunctional catalysts can also be used to help improve the reaction rate of hydrogen production to increase the hydrogen yield. The proposed structure of such bifunctional catalysts has been shown in Figure 10.

Of all the catalysts studied for the gasification process, alkali metals were the first to be researched (Francke et al., 2018). Unsupported catalysts based on natural minerals, noble gas, and synthetically available triggers have also been studied and developed for hydrogen production via direct biomass gasification (Al-Rahbi and Williams, 2017; Yang et al., 2019). These catalysts can undergo rapid action, decrease the amount of tar production, and increase total hydrogen gas. Sometimes even microorganisms are used that are chemically active. Such microbes tend to generate electricity by producing electrons. When these organisms are used as “catalysts,” the process becomes very economical and highly efficient. Electrochemically active microbes are used in a cell with protons, electrons, and other chemical substances to ease the reaction. Because of the coupling of such components, direct production of hydrogen occurs (Kim et al., 2017; Ma et al., 2021). The dissolved organic components get oxidized, and the protons are reduced to give hydrogen.

Metals like nickel, iron, and cobalt have also been used in methane pyrolysis reactions without the need for a supporting structure (Srilatha et al., 2017). This is because of their enhanced activity, temperature conditions, and ability to produce solid carbon as the byproduct. These metal catalysts also allow solubility and diffusion of carbons through catalysts’ surface. Of all the catalysts mentioned above, Ni has the highest activity and least toxicity for this reaction (Ouyang et al., 2019). However, these elements undergo deactivation at very high temperatures (>600°C). In the case of unsupported catalysts, the catalytic activity during aqueous phase reforming was noted to be (He et al., 2013):

Platinum = nickel > ruthenium > rhodium = palladium > iridium

Of all the catalysts, Ni has shown to display the lowest reformation of the reactants and allows for efficient hydrogen production. Alkali metals derived from acids have also shown efficiency during the catalytic process – like the alkali metal salts derived from weak acids. These include potassium carbonate, potassium sulfide, sodium carbonate, and sodium sulfide. During these reactions, lower temperatures are better preferred, that is, between 700 and 925°C (Mei Wu et al., 2014). These catalysts are added to the gasifier present on coal or char. These catalysts, however, are not quickly recovered after the gasification process. Moreover, elements present in the catalyst, like sulfur, can sometimes poison the catalysts during the reaction.

### Metal Oxide Supported Catalysts

Various transition metals supported on oxides are also used as catalysts in TMD reactions (Shen and Lua, 2015a). These include metals like Ni, Fe, and Co, and others. Various studies (Khan et al., 2016; Abdullah et al., 2017; Chen et al., 2020b; Baig et al., 2021) have shown the effects of different metal oxide supported catalysts like La_2_O_3_, ZiO_2_, Al_2_O_3_, SiO_2_, and TiO_2_ involved in producing hydrogen (Kim et al., 2011; El Hakim et al., 2021). In a study by Ibrahim *et al.*, it was found that Fe over La_2_O_3_ showed promising results for TMD at temperatures ranged between 500 and 700°C. Another study by Shen and colleagues on Ni supported on TiO_2_ revealed that this catalyst showed higher stability and expressed a high hydrogen production rate as compared to a catalyst unsupported by Ni (Shen and Lua, 2015b). Of all studies on metal oxides supported on carbon substances, silica-based materials serve as better catalysts because of their increased surface area. They also possess enhanced thermal stability, changeable pore size, and increased diffusion ability (Di Michele et al., 2019). This makes them an ideal candidate to be used in metal oxide supported catalysts for hydrogen production.

The behavior of metal oxide supported catalysts has shown to be far more convenient and useful in POM reactions. Ni-based oxide supported catalysts like the ones kept on Al_2_O_3_, La_2_O_3_, ZrO_2_, CeO_2,_ etc. are developed through solid-state reaction and wet impregnation. These catalysts, however, are easily deactivated because of the rapid deposition of carbon. Moreover, contamination because of Ni nanoparticles may also result in their deactivation. However, the metal catalysts that are supported on ceria and alumina have shown to be very promising. This is because of the large surface area of the Ni nanoparticles on the alumina surface and the available spots in ceria structures that allows for the direct adsorption on those sites (Singha et al., 2017).

On the contrary, catalysts attached to Mg-based structures that rapidly undergo deactivation due to the creation of NiO/MgO irreducible complexes in the solution. Metal oxides like La_2_O_3_ allow for decreased carbon formation because of the high scattering of Ni nanoparticles (Kumar et al., 2019). Moreover, these structures also cause lanthanum oxycarbonate development that causes further gasification of carbon structures. ZrO_2_ also plays an essential role in serving as the support structure for metal catalysts. However, these oxides reduce the oxygen presence that is essential in POM reaction (Hongloi et al., 2019).

Among the metal oxide supported catalysts that are well studied, Ni/Al_2_O_3_ has gained wide popularity because of its cheap availability and high stability (Alvarez-Galvan et al., 2019). Other metal oxide supports include MgO, SiO_2_, TiO_2_, CeO_2_ etc. Furthermore, Al_2_O_3_ disallows catalyst frittage and increases the overall mechanical stability of the catalysts. Beyond 973K, the sensitivity of the Al_2_O_3_ based metal oxide catalysts also increases. Hence, any changes in Al_2_O_3_ support create catalysts that directly affect the catalysts (Koh et al., 2007). In some instances, transition metal oxides like CeO_2_, ZrO_2_, and Cr_2_O_3_ are used along with alumina components (Silva et al., 2012). However, oxygenates rapidly undergo dehydration and give ethylene, resulting in carbon deposition and catalyst deactivation (Ali et al., 2016; Rogers et al., 2016). Studies have shown that CeO_2_ support increases the stability and activity of Ni-based catalysts (Arora and Prasad, 2016). Moreover, the addition of Ce to ZrO_2_ results in strong interaction between the individual substances present on the catalyst. Also, Ce causes enhanced storage capacity of oxygen and allows for the synthesis of transferrable oxygen during the reaction process. Hyun Seog Toh and colleagues studied multiple metal oxide supported catalysts (like MgO, Ce-ZrO_2_, MgAl_2_O_4_) however, Ni catalysts supported on MgAl_2_O_3_ showed the maximum efficiency and stability (Roh et al., 2001; Shang et al., 2017).

Metal oxides of alkaline earth metals are favorable for gasification reaction because they permit reformation reactions (Nikolaidis and Poullikkas, 2017). When metal catalysts like Cu catalysts are introduced into the reaction, additional elements like promoters are also used. This is to decrease the amount of non-required products (like the soot and tar) and increase the total hydrogen produced. The metal oxides, including the oxides of transition metals, are alkali earth metals are often introduced as the promoters during the reaction (Gupta et al., 2011; Qu et al., 2020).

The ZnO/Zn redox pairs have shown to have better superiority among other metal oxide supported catalysts during hydrogen production via this reaction (Steinfeld, 2002). However, this metal oxide pair is not feasible for the large-scale production of hydrogen. Moreover, efficient separation of the products poses a challenge (Koepf et al., 2016). Hence, because of the shortcomings, CeO_2_ is a better candidate for water splitting reaction and hydrogen production (Gokon et al., 2013). However, these catalysts function at high temperatures of 1700–3000 K. Other metal oxide supported catalysts include the redox pairs of Mn_2_O_3_/MnO, SnO_2_/SnO, Fe_2_O_4_/Fe, etc. The enhanced surface area of platinum supported on Al_2_O_3_ and ZrO_2_ also shows very high activity. However, these catalysts exhibit rapid deactivation too. Platinum catalyst supported on TiO_2_ also show very high stability. However, they too get deactivated when used for prolonged durations.

Fe catalysts supported on other metal oxides like Al_2_O_3_ based catalysts have been studied for this reaction and have shown promising results (Zhou et al., 2016). When such catalysts are used, they offer *in situ* catalysis during methane pyrolysis. This was seen in the work put forward by Yeheskel and coworkers (Yeheskel and Epstein, 2011). The authors discussed when Fe(CO)_5_ and Fe(C_2_H_5_)_2_ are decomposed. Fe clusters are produced during the pyrolysis reaction. Besides, various other gases are also produced as by-products. These gases produced, however, undergo further decontamination steps.

Metal oxide supports like Al_2_O_3_ and Fe_3_O_4_ have shown to be very efficient in hydrogen synthesis via aqueous steam reforming (Gumina et al., 2019). Valenzuela and colleagues were the first teams who developed this process for hydrogen production (Valenzuela et al., 2006). They employed Pt catalysts supported on Al_2_O_3_. High activity of the catalyst structure and large hydrogen production was noted during the reaction. In another study, the catalytic activity of CeO_2_-ZrO_2_ and CeO_2_-TiO_2_ was studied (Chen et al., 2017a). It was noted that both sets of catalysts showed high activity and efficiency. Moreover, these catalysts, including Pt-Al_2_O_3,_ were observed to show high catalytic activity and were recyclable.

Among the metal oxide supported catalysts, K_2_CO_3_ has shown high catalytic activity during the gasification reaction compared to Ni catalysts supported on metal oxides. It has been observed that hydrogen production is increased two times when K_2_CO_3_ is used as a catalyst (Li et al., 2010). This could be because of the high and uniform solubility of the catalyst in the reaction mixture. Other catalysts like SiO_2_, KAlSiO_4_, NiO, and KAlSi_3_O_8_ have been shown to undergo rapid deactivation owing to large carbon deposits on their surface. Other metal oxide-supported catalysts like ZnO were shown to exhibit quick activity in contrast to SnO_2_ catalysts; the hydrogen yield was also enhanced.

### Noble Metal-Based Catalysts

Noble metal catalysts like ruthenium and rhodium used in the SMR process exhibit increased activity and high stability (Chen et al., 2017b). Other noble metals like platinum and iridium show enhanced electrocatalytic activity and can be attached to other elements to give better catalytic activity (Swain et al., 2018). However, there are a few drawbacks to these catalysts including their high cost of production and instability (Digraskar et al., 2019). These can be overcome by reducing the size of the noble metals attached to porous carbon structure. This increases the overall efficiency of catalysts. Furthermore, they can be attached to other transition metals to enhance the catalyst’s activity (Li et al., 2019). These steps can be incorporated at an industrial level to reduce their overall cost of production.

Noble metals like palladium, rhodium, platinum, iridium also serve as noble metal-based catalysts in POM reaction (Figen and Baykara, 2018; Ma et al., 2019). Studies have shown the reducing nature of noble metals like ruthenium that results in lanthanide oxide-supported ruthenium structures that are used catalysts in POM (Lian et al., 2018). Owing to the enhanced performance of noble metal catalysts, these catalysts have gained a great deal of attention from the scientific community. In a study by Alvarez-Galvan and colleagues, it was discovered that rhodium, when used as the supporting material of metals like Ni, promotes the further reduction of the catalyst, thereby improving the overall catalytic activity during POM (Alvarez-Galvan et al., 2019). It has been noted that the catalytic activity of ruthenium-based catalysts is significantly greater at greater flow rates than Ni-based catalysts (Wysocka et al., 2019). However, the method used to prepare the catalyst and the supporting structure also determines their activity (Horn and Schlögl, 2015).

Noble metal-based catalysts show a better and bigger ratio of hydrogen and CO than metal oxide supported catalysts. For instance, when present in the Ni-based catalyst, palladium disallows the oxidation of catalyst and maintains its reduced state. The bigger the noble metal clusters of the catalyst, the stronger the interaction and, hence, the more stable the catalyst’s overall structure (Mosinska et al., 2020). This was seen in the study by Keiichi Tomishige and coworkers. The authors reported that Ni and Pt catalysts restrict the formation of carbon structures on the catalysts. In another study, Antonio Vita and coworkers studied CeO_2_ based catalysts attached to metals like rhodium, platinum, nickel used in autothermal reforming reactions (Vita et al., 2015). Of all the catalysts studied by the authors, catalysts based on CeO_2_ showed the least stability, while catalysts based on Ru showed very high methane conversion rates and high hydrogen yields.

Rhodium based catalysts are the most popularly used noble metal-based catalysts for the gasification process. This is because of the enhanced selectivity and cost ineffectiveness. However, these are relatively quite expensive. Regardless, noble metal-based catalysts, including rhodium, palladium, and platinum, among others, have been found to have enhanced activity, stability, and increased resistance to deposition of carbon on the catalysts’ surface (Hakouk et al., 2018). Among the noble metal-based catalysts, platinum has shown the maximum efficiency for the water-splitting reaction. These elements act as electron magnets and decrease the potential for hydrogen production (Wang et al., 2015). When supported on TiO_2_, electron transfers occur from TiO_2_ to platinum. On the contrary, noble metals like rhodium do not show very efficiency during this reaction, and hence the amount of hydrogen production is lowered (Jana et al., 2010).

Like the metal-based catalysts, the noble metal-based catalysts show better activity and stability than metal catalysts without supporting materials. This was seen in the work put forward by Pudukudy and colleagues (Pudukudy et al., 2018). They developed techniques that could synthesize hydrogen devoid of carbon-based co-products. They used ceria along with platinum-based nickel catalysts. The authors noted that there were no separate peaks in regards to platinum observed. This meant that the addition of the noble metal platinum permitted good dispersion on the catalyst surface. Moreover, the overall activity was noted to be highly efficient in the addition of platinum. Hence, such noble metal-based catalysts are also considered to increase the catalyst’s overall activity. Various kinds of catalysts that are used in hydrogen production by methane pyrolysis are shown in Table 6.

**TABLE 6:** Various catalysts used in hydrogen production by methane pyrolysis.

Catalyst type	Examples
Metal catalyst	Ni, Fe, Co
Metal supported catalysts	NPs, MOFs, Carbon NMs
Non supported catalysts	Cellulose, ionic liquids
Metal oxide supported catalysts	La_2_O_3_, ZiO_2_, Al_2_O_3_, SiO_2_
Noble metal supported catalysts	Pt, Ir, Pd, Rh, Ce

A few years after the first APR activity for hydrogen production was recorded, Erbatur and colleagues then investigated APR using various catalysts. They noted that platinum displayed the best catalytic efficiency and activity during the reaction (Meryemoglu et al., 2010). After platinum, ruthenium, and palladium recorded the highest activity and catalytic efficiency. This could be because of the availability of strong minerals that are essential for hydrolytic activity. Moreover, the mineral acids result in the drainage of the noble metals into the solution. This thus limits the recycling of the catalysts. However, their high expense is the sole disadvantage of using noble metal-based catalysts.

Ruthenium based catalysts have shown five to ten times better activity than other noble metal-based catalysts (Molino et al., 2018). The only drawback is the high rate of deactivation of ruthenium-based catalysts because of the carbon supporting material often used along with this catalyst. During the reaction, carbon is also shown to be utilized, which reduces ruthenium activity. Other elements, like platinum and palladium, have also shown promising catalytic activity. They can overcome the drawbacks posed by nickel-based catalysts. Their only drawback is their expensive availability. Tomishige and coworkers discovered that the order of catalyst activity to be (Tomishige et al., 2004):

rhodium > palladium > platinum > nickel = ruthenium

Therefore, among most catalysts, noble metal-based catalysts show the maximum activity during coal gasification reaction. Many studies have carried out to understand the catalytic process of the various techniques. Various kinds of catalysts have been used for hydrogen production via the TMD process. Some of these are comprehensively discussed below in Table 7 **.**

**TABLE 7:** Catalysts used for hydrogen production via TMD process.

Catalyst	Catalyst type	Methane conversion (%)	Hydrogen yield	Temp (°C)	Methane flow rate	References
NiO/Al_2_O_3_–SiO_2_	Metal oxide catalyst	∼40	1,730 (mol H_2_/mol Ni)	550	30 cc/min	Ashok et al. (2008a)
Activated carbons	Non supported catalyst	—	—	900	20 ml/min	Ashok et al. (2008c)
Fe	Non supported metal catalyst	∼80	—	800	27.5 N cm^3^/min	Cunha et al. (2008)
Co	Non supported metal catalyst	∼80	—	800	27.5 N cm^3^/min	Cunha et al. (2008)
Ni	Non supported metal catalyst	∼80	—	800	27.5 N cm^3^/min	Cunha et al. (2008)
FeMo/MgO	Metal oxide supported catalyst	87	—	900	50 ml/min	Pinilla et al. (2011)
Ni/SiO_2_	Metal oxide supported catalyst	35	3.4 (mol H_2_/mol CH_4_)	550	—	Zhang and Amiridis, (1998)
Ni/SiO_2_–Al_2_O_3_	Metal oxide supported catalyst	∼80	—	700	20 cm^3^/min	Suelves et al. (2005)
Ni/MgO	Metal oxide supported catalyst	35–40	—	550	60 N cm^3^/min	Bonura et al. (2006)
Ni/SiO_2_	Non supported catalyst	35–40	—	550	60 N cm^3^/min	Bonura et al. (2006)
Ni/Cu–Nb_2_O_5_	Metal supported catalyst	∼45	7,274 mol H_2_/mol Ni	600	40 cm^3^/min	Li et al. (2004)
Ni/Cu–alumina	Metal supported catalyst	70	—	750	68 cm^3^/min	Li et al. (2000)
Ni/Cu–Si	Metal supported catalyst	∼80	—	700	20 cm^3^/min	Lázaro et al. (2007)
Ni/Cu–MgO	Metal oxide supported catalyst	45	—	700	60 cm^3^/min	Wang and Baker, (2004)
Ni/Cu–Al	Metal supported catalyst	65	—	700	20 cm^3^/min	Suelves et al. (2006)
Ni/Cu–Mg	Metal supported catalyst	∼67	—	700	20 cm^3^/min	Moliner et al. (2008)
Fe/MgO	Metal oxide supported catalyst	∼87	—	800	50 ml/min	Pinilla et al. (2011)
NiCuAl	Metal supported catalyst	31.2	—	700	150 N cm^3^ min^−1^	Suelves et al. (2009)
Co/Al_2_O_3_	Noble metal supported catalyst	∼22	—	700	35 cm^3^/min	Nuernberg et al. (2008)
Fe/Al_2_O_3_	Metal oxide supported catalyst	7.9	—	625	—	Avdeeva et al. (2002)
NiO-CuO	Non supported catalyst	85	—	750	25 ml/min	Lua and Wang, (2013)
Ni/SiO_2_	Non supported catalyst	88	∼89%	750	30 ml/min	Saraswat and Pant, (2013)
Ni–Mo/Al_2_O_3_	Metal oxide supported catalyst	∼80	∼88%	750	—	Awadallah et al. (2013)
Al_2_O_3_–TiO_2_	Metal oxide supported catalyst	∼70	59%	700	50 sccm	Awadallah et al. (2014)
Ni-Co/Al_2_O_3_-MgO	Metal oxide supported catalyst	∼80	—	800	500 ml/min	Karaismailoglu et al. (2019)
Ni	Non supported catalyst	53	—	700	70 ml/min	Li et al. (2017)
Magnesium-NiO	Metal oxide supported catalyst	82	1.8%	800	30 sccm	Al-Fatesh et al. (2020)
Ni_3_Si_2_O_5_(OH)_4_	Non supported catalyst	85	—	800	—	Zhang et al. (2019b)
NiO-MgO-Al_2_O_3_	Metal oxide supported catalyst	91	—	800	—	Chai et al. (2017)
Ni-La@KCC-1	Metal supported catalyst	96	—	750	100 ml/min	Abdulrasheed et al. (2020)
Ni/Ce-ZnAl2O4	Noble metal supported catalyst	82	92%	800	10°C/min	Movasati et al. (2017)
Ni/alumina-zirconia	Metal supported catalyst	90	72%	800	32.5 ml/min	Fakeeha et al. (2020)
Ni/SiO_2_Al_2_O_3_	Metal oxide supported catalyst	100	—	900	25 ml/min	Ali et al. (2016)
Ni/MgAl_2_O_4_	Metal oxide supported catalyst	75	—	850	—	Katheria et al. (2016)
Ni/Ni_2_Al_2_O_5_	Metal oxide supported catalyst	82	∼20%	∼650	—	Rogers et al. (2016)
Ru/ZnLaAlO_4_	Noble metal supported catalyst	72	99.8%	800	50 N ml/min	Khani et al. (2016)
Ni/CeO_2_/Al_2_O_3_	Noble metal supported catalyst	∼94	42.52%	∼900	—	Peng et al. (2017)
Ni/Ru/γ-Al_2_O_3_	Noble metal supported catalyst	80	—	∼650	—	Calzada Hernandez et al. (2020)
Ni/Pt- γ-Al_2_O_3_	Noble metal supported catalyst	80	—	∼700	—	De Vlieger et al. (2012)

## Catalytic Deactivation

We have discussed a number of catalysts above. Though the metal catalysts and metal-based catalysts show an enhanced conversion during the TMD reaction, its rates of deactivation is also high. The biggest challenge that causes deactivation is the development of metal carbide structures over the catalysts surface. Also, sulfur content also poses a significant concern during the process. These sulfur components also cause poisoning of the catalysts. Unlike the carbon-based catalysts, metal-based catalysts cannot absorb these sulfur components from the feedstock. This limited life of the catalyst upon deactivation couple with lowered hydrogen to carbon ratio slows the industrial applicability of such catalysts. This therefore causes the development of low quality fuels that possess low heating value when compared to fossil fuels (Chew and Bhatia, 2009; Alaba et al., 2016).

**FIGURE 11:**
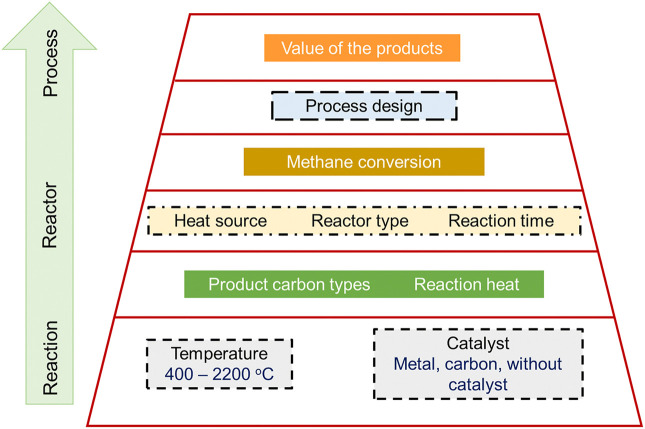
Catalytic decomposition pathway during TMD process. Adapted with permission from reference Hasnan et al. (2020b), copyright@2020 (Springer).

Catalyst regeneration, in turn, would be done by burning off carbon deposited that affects the entire life of the catalysts (Cao and Yu, 2016). When carbon-based catalysts are used in the reaction, there are many advantages like more flexibility in the reaction, and lack of poisoning due to other elements. Moreover, these catalysts display lower resistance to temperature changes. Figure 11 displays the general pathway followed for catalyst selection during TMD process.

The main reason behind any catalyst’s deactivation is the deposition of solid carbon/coke on the catalyst’s surface. This carbon lowers the activity function and hence hampers the functionality of the catalyst. Studies have suggested the Ni-based catalysts show high efficiency during TMD. The reaction is carried out at temperatures ranged between 500 and 600°C. This temperature range is not as high as required to attain equilibrium. However, at increased temperature ranges, Ni catalysts undergo rapid deactivation. Hence, sometimes Fe is used instead of Ni. This is because Fe unlike Ni can stand through high temperatures without undergoing deactivation. Moreover Fe is cheaper than Ni (Qian et al., 2020).

For TMD to proceed efficiently and for the catalysts to remain active for prolonged durations, better alternatives for catalysts have to be studied. Another challenge during TMD is the poisoning of the catalysts because of sulfur compounds. These poisons also deactivate the catalysts and hinder the reaction progression. Furthermore, the metal-support made available to the catalyst also plays a significant role in catalytic deactivation. If the support and metal bond are not strong, the metal counterpart’s detachment can also cause the catalyst’s deactivation. When the metals get detached, they tend to get localized in nanotubes. This causes the deactivation of the catalyst over a while. When the bonds between metal and support are more robust, the catalyst deactivation is slowed; however, catalyst deactivation follows despite the prevailing circumstances.

## Catalyst Regeneration

A catalyst cannot maintain its functionality and selectivity for prolonged durations. Their activity reduces with time, which results in its deactivation. This deactivation may sometimes occur rapidly and sometimes may take months (like sulfur poisoning cases). Sometimes, catalytic deactivation can also take years, like in ethylene hydrogenation and ammonia production. Catalytic poisoning, coking, and restructuring of the catalyst are responsible for the catalytic deactivation. After catalyst deactivation, catalysts can be regenerated using oxygen (Saad and Williams, 2016; Gao et al., 2019; Vasiliades et al., 2020). The significant methods employed for catalyst regeneration include combustion and gasification. Oxygen is considered during regeneration via combustion, while steam is used during regeneration via steam. The following two stoichiometric reactions highlight the regeneration of catalysts (Srilatha et al., 2017):

$$\begin{array}{l} {\text{C }+\text{ O}}_{2}\;\to {\text{ CO}}_{2}\\ \Delta {\text{H}}_{1073}\;=\;-394\text{.}7\text{ kJ/mol} \end{array}$$

$$\begin{array}{l} {\text{C }+\text{ H}}_{2}\text{O }\to {\text{ CO }+\text{ H}}_{2}\\ \Delta {\text{H}}_{1073}\;=\;135\text{.}9\text{ kJ/mol} \end{array}$$

$$\begin{array}{l} {\text{C }+\text{ CO}}_{2}\;\to \;2\text{CO}\\ \Delta {\text{H}}_{1073}\;=\;174\text{.}5\text{ kJ/mol} \end{array}$$

Catalytic activation is done by removing the carbon deposition from the catalyst’s matrix sites, which increases the total surface area of the catalysts for further reactions. However, during catalyst regeneration, purification steps became indispensable to get pure hydrogen as the end product. This is because regeneration steps result in the production of CO_x,_ which makes the purification step necessary. Some catalysts, however, do not require regeneration steps and consideration activity. Examples of such catalysts include nickel supported on ZrO_2_ and Al_2_O_3_ (Shang et al., 2017). Over time, these catalysts have physically blocked the reactor and cause a drop in pressure throughout the catalyst bed. Hence, catalyst regeneration becomes a necessary step to avoid inefficiency in the catalytic activity during the thermal decomposition of methane for hydrogen production.

Catalytic regeneration has been observed in the works put forward by Amin and colleagues (Amin et al., 2012). They introduced three different Ni-based catalysts in a fluidized bed reactor at a temperature above 500°C. They used oxygen as the activating agent for the reaction to proceed. The authors noted that carbon was removed entirely on all the catalysts studied. In another similar study, Amjed and coworkers studied activated carbon during TMD above 900°C (Al-Hassani et al., 2014). The authors developed models to understand the functional surface area in the catalyst and the volume of pores. This kinetics was designed to understand the process of catalytic deactivation better. Moreover, it was also observed that rapid deterioration of the catalysts occurred at very high temperatures due to catalyst frittage or weakening.

## Conclusion and Future Perspective

Production of hydrogen devoid of CO_x_ components is particularly challenging; however, it’s essential for eco-friendly measures. In this review, we have discussed the various kinds of catalysts available (including metal-based catalysts, noble-metal-based catalysts, non-supported catalysts, metal-oxide-based catalysts) and the mechanism of different processes available for hydrogen production. Though the thermocatalytic decomposition of methane and the catalysts involved have been thoroughly studied, factors like optimization of the catalysts, cost of the process, and the necessity of regeneration of catalysts still require in-depth exploration and analysis. Moreover, despite alternative options being available, the dependence on fossil fuels and their derivatives will continue even in the future. These options, however, do not limit emissions of greenhouse gases. Hence additional steps can be added in the process such that carbon black is produced. These compounds are valuable in the market; therefore, the overall expense of the process gets reduced. Other external factors, like supporting conditions and the kind of catalysts used, also influence the cost and carbon type formed as a by-product.

Studies show that thermocatalytic decomposition of methane for hydrogen production is also affected by the temperature at which reactions proceed and even the type of catalysts used. For instance, Fe-based catalysts showed high activity at high-temperature conditions. Nickel-copper alloy catalysts show that supported catalysts have better functions as compared to unsupported catalysts. However, their rapid deactivation because of carbon deposition on the catalysts’ surface serves as a limiting factor of the process. Works of literature have also shown that carbon-based catalysts show enhanced activity because of their cheap availability, stability at high temperatures, and resistance to sulfur poisoning. More research needs to be carried out to study other existing materials that can serve as better catalysts and resist catalytic deactivation during hydrogen production processes. Besides, better-supporting structures will have to be researched so that the resulting catalysts have the higher surface area and increased porosity during catalysis. Promoters can be used to increase the stability of the catalyst. For instance, promoters like CeO_2_ can improve the stability of active sites and improve the catalysts’ functionality. Other structures like mesostructured silica nanoparticles can also be used as a supporting structure because of their vast surface area and changeable pore size. Moreover, they exhibit high resistance to heat and show increased mechanical stability.

So far, Ni and Fe are popular options among the available catalysts owing to their inexpensive availability and high activity. More importantly, Ni-based catalysts were found to more reliable than others. However, these catalysts show limitations because of strict temperature requirements. Hence these catalysts are linked to other elements like Cu or Fe to enhance the stability and overall activity.

## Abbreviations

TMD, Thermo catalytic methane decomposition; Kmf, Threshold constant; SMR, Steam methane reforming; WGS, Water-gas shift reaction; POM, Partial Oxidation of Methane; ATR, Autothermal Reforming; DBG, Direct Biomass Gasification; TWS, Thermochemical Water Splitting; MP, Methane pyrolysis; CSS, CO2 capture and storage; APR, Aqueous Phase Reforming; CG, Coal Gasification.
